# Opposing Effects of Expectancy and Somatic Focus on Pain

**DOI:** 10.1371/journal.pone.0038854

**Published:** 2012-06-19

**Authors:** Natalie E. Johnston, Lauren Y. Atlas, Tor D. Wager

**Affiliations:** 1 Department of Neuroscience, Weill Cornell Medical College, New York, New York, United States of America; 2 Department of Psychology, New York University, New York, New York, United States of America; 3 Department of Psychology and Neuroscience, University of Colorado, Boulder, Colorado, United States of America; University of Manchester, United Kingdom

## Abstract

High-pain expectancy increases pain and pain-related brain activity, creating a cycle of psychologically maintained pain. Though these effects are robust, little is known about how expectancy works and what psychological processes either support or mitigate its effects. To address this, we independently manipulated pain expectancy and “top-down” attention to the body, and examined their effects on both a performance-based measure of body-focus and heat-induced pain. Multi-level mediation analyses showed that high-pain expectancy substantially increased pain, replicating previous work. However, attention to the body *reduced* pain, partially suppressing the effects of expectancy. Furthermore, increased body-focus had larger pain-reducing effects when pain expectancy was high, suggesting that attempts to focus on external distractors are counterproductive in this situation. Overall, the results show that attention to the body cannot explain pain-enhancing expectancy effects, and that focusing on sensory/discriminative aspects of pain might be a useful pain-regulation strategy when severe pain is expected.

## Introduction

Recent studies have found that expectations about pain intensity and pain-mitigating treatments modulate pain reports and brain responses to noxious stimuli [1]–[5]. In a recent study [6], for example, auditory cues that signaled high (vs. low) upcoming pain substantially increased pain reports; these effects were formally mediated by noxious heat-evoked responses in rostrodorsal anterior cingulate, thalamus, and left anterior insula–the brain regions most closely associated with pain experience (e.g., [7], [8]). While expectancies are increasingly recognized as meaningful influences on nociception, the psychological processes that constitute expectations and mediate their effects on pain (and other affective events) have not been well described.

Expectancies may work by influencing attention, affective appraisals, or other processes. Expectations about the environment drive visual attention [9], [10]; likewise, high-pain expectancy may redirect endogenous “top-down” attention toward pain [11] and/or enhance “bottom-up” vigilance, both of which could intensify pain experience. Conversely, low-pain expectations could act as a safety signal, allowing attention to be directed elsewhere. As distraction–the diversion of attention–can have large and reliable effects on experimental pain [12], and can also influence the processing of pain-related signals in the cortex [13]–[15], attention to or away from the body might mediate the cue-based expectancy effects on pain. Alternatively, expectancies may work by altering the meaning or *appraisal* of sensory information. In Gross’s model of emotion regulation, for example, attention to and appraisal of emotional events are separate processing stages [16]. If this is the case for pain expectancy, expectancy effects should be independent of the effects of attention to the body. Consistent with this, recent work suggests that distraction and placebo treatments both reduce pain, but that they do so with additive effects, suggesting that they rely on separate mechanisms [17]. While this suggests that placebo-based expectancy effects are independent from attention, cue-based threat of pain (i.e. expectancy for high vs. low pain) might interact with attention to influence pain perception. Thus, the relationship between expectation and attention as modulators of affective processes remains unclear.

The current study was designed to directly address this question by independently manipulating pain expectancies and top-down attention toward the body, or somatic focus. As in Atlas et al. [6], we manipulated expectancy with auditory pain-predictive cues, and examined effects of these cues on pain evoked by noxious thermal stimulation, holding constant the stimulation temperature. To measure the degree of attention to vs. away from the body (i.e., *somatic focus*) during pain, participants performed two concurrent tasks while experiencing pain: a *heat discrimination task (HDT)*, involving judgments about whether the stimulus temperature increased or decreased slightly, or remained constant, and a *visual discrimination task* (*VDT*) that involved the identification of briefly presented, masked letters. Performance on the HDT provided a measure of somatic focus. Finally, we manipulated monetary payoffs across trials to favor accurate performance on either the HDT or VDT, which allowed us to manipulate somatic focus.

**Figure 1:**
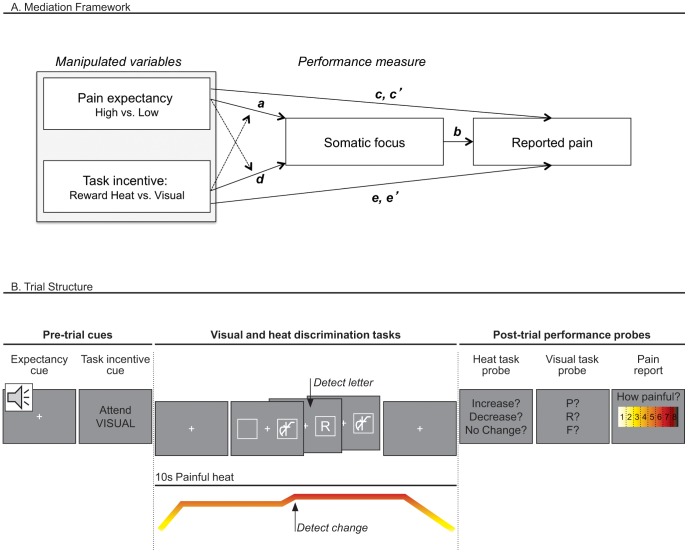
Mediation analysis framework and experimental design. A. **Mediation framework.** We manipulated expectancy with cues that predicted high or low pain (“Pain expectancy”) and attention using cues that referred to performance-based rewards (“Task incentive”). We tested cue effects on pain evoked by noxious thermal stimulation, holding constant the stimulation temperature (Path *c* and Path *e*). The first mediation model tested effects of Expectancy Cue (controlling for Task-Incentive Cue) on somatic focus (Path *a*); and on pain (Path *c*) when controlling for somatic focus (Path *c*’). The second model tested the effects of Task-Incentive Cue (controlling for Expectancy Cue) on somatic focus (Path *d*); and on pain (Path *e*), when controlling for somatic focus (Path *e’)*. Each model tested whether Somatic Focus influences pain, controlling for Expectancy and Task-Incentive Cues (Path *b).* We also tested for interactions between Task incentive and Pain expectancy effects on somatic focus (dashed lines). B. **Trial Structure.** Participants received two preparatory cues on each trial: 1) an auditory *pain expectancy cue* that signaled the probable level of pain (low vs. high) to expect on that trial; and 2) a visual *task incentive cue* (“Attend HEAT or Attend VISUAL”) directing them to attend preferentially to one of two simultaneous tasks that occurred during thermal stimulation: a somatic, *heat discrimination task*, which drew attention toward the thermal stimulus on the body, and a *visual discrimination task*, which drew attention toward a visual stimulus on a computer screen and away from the body. In the heat discrimination task, participants were informed that the intensity of the pain might increase slightly, decrease slightly, or not change from the target temperature on each trial. In the visual discrimination task, a letter was briefly presented and masked immediately before and after by overlapping letters. After the noxious thermal stimulation ended, participants were prompted to report both the letter presented on each trial and the temperature change, as well as their subjective pain rating.

These manipulations allowed us to test whether the effects of expectancy cues on pain are mediated by changes in somatic focus. As diagrammed in Figure 1A, the mediation model we used consists of tests that address three critical questions: 1) Do cues that predict high vs. low upcoming pain influence somatic focus (Path *a* in Figure 1A)? 2) Do changes in somatic focus influence pain experience (Path *b*)? and 3) Are cue effects on pain mediated by changes in somatic focus? This latter mediation effect tests whether cue effects on pain (Path *c*) are significantly reduced when controlling for somatic focus (Path *c*’) (thus, the significance of the difference [*c – c*’]). We tested these relationships using multi-level mediation [6], [18]–[20], as all variables were manipulated or measured within individuals.

This design also allowed us to ask basic questions about the effects of somatic focus and potential interactions between expectancy and task incentive (shown by dashed arrows in Figure 1A). First, it allowed us to test whether increased somatic focus did indeed increase pain, or whether it had the opposite effect. On one hand, somatic focus could increase pain: Distraction often reduces pain [17], [21], and anticipatory pain-related activity is associated with increased pain unpleasantness and reduced performance on other tasks [22]. On the other hand, top-down attention to the body with a focus on the non-affective, sensory/discriminative aspects of pain could actually reduce pain in at least two ways. First, it could promote positive appraisal or acceptance strategies, and second, it could compete with the ‘hot,’ affective representation of pain [23]. Additionally, the effects of increased somatic focus might be different when pain expectancy is high vs. low, i.e., somatic focus and expectancy may interact. For example, the threat of high pain may promote anxious or fearful thoughts, which may result in fewer cognitive resources toward distractors [24]. If this is the case, then distraction by attending to another modality may be counterproductive when pain expectancy is high.

## Results

### Expectancy Effects on Pain and Relationship to Somatic Focus: Mediation Model 1

**Figure 2:**
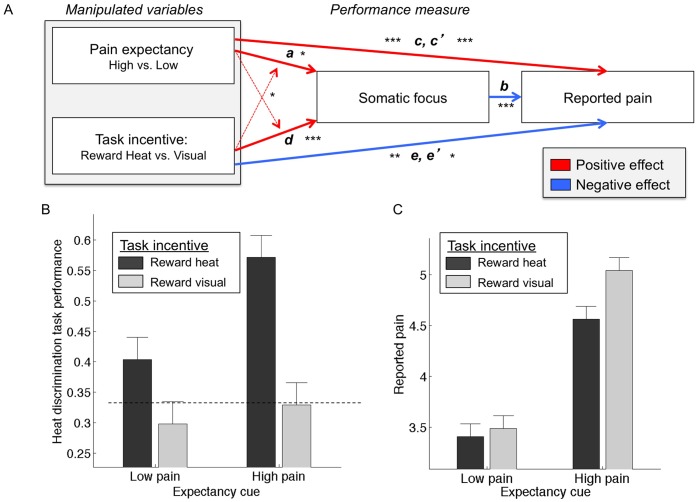
Expectancy and task incentive effects on pain and somatic focus. A. **Mediation Model Results:** There were main effects of: 1) Expectancy Cue (high vs. low) on Somatic Focus (Path *a*); 2) Somatic Focus on pain ratings (Path *b*); 3) Expectancy cue on pain ratings (Path *c*); 4) Task-Incentive Cue on Somatic Focus (Path *d)*; and 5) Task-Incentive cue on pain ratings (Path *e*) (see below for details of main effects; full statistics are presented in Table 1). Expectancy effects on pain reports were mediated by changes in somatic focus (*c – c’*) with a negative mediation effect, indicating that increases in somatic focus partially suppressed the pain-intensifying effects of high pain expectancy (p<0.05). The effect of Task-Incentive cue on pain was mediated by changes in somatic focus (*e – e’)* (p<0.05). These results indicate that incentivizing the heat discrimination task reduced pain by increasing somatic focus. B. **Heat discrimination performance as a function of Task-Incentive Cue and Expectancy Cue.** Main effect of Task-Incentive Cue on Somatic Focus (corresponding to Path *d*): Participants performed at chance (dashed line) when visual task was incentivized, and above chance when heat-discrimination task was incentivized (p<0.001). Main effect of Expectancy Cue (high vs. low) on Somatic Focus (Path *a*): participants performed better on the heat discrimination task when they expected High pain (p<0.05). There was a significant interaction (dashed arrows in Figure 2A) between Task-Incentive Cue and Expectancy Cue, such that heat discrimination task performance was better under High pain expectancy (p<0.001), while effects under Low pain expectancy were marginally significant (p = 0.03 one-tailed). C. **Pain ratings as a function of Task-Incentive Cue and Expectancy Cue.** There was a main effect of Expectancy Cue (corresponding to Path *c*), such that participants reported less pain when medium heat was preceded by low-pain cues, relative to high-pain cues (p<0.001). There was also a main effect of Task Incentive Cue (Path *e*), such that participants reported less pain during moderate-pain trials when they were preferentially rewarded for performance on the heat discrimination task, relative to the visual discrimination task (p<0.01). There was also a main effect of Somatic Focus (Path *b*), such that somatic focus reduced pain (p<0.001).

Our first path model, presented in Figure 1A and Figure 2A, tested whether Expectancy cues directly affect pain reports (Path *c)*, and whether this relationship is mediated by changes in somatic focus (*c-c’)*. We controlled for Task-Incentive cue in all paths. Statistics for each analysis are presented in Table 1. As shown in Figure 2A, there was a significant effect of Expectancy cue (high vs. low) on pain reports (Path *c* in Figure 2A and Table 1) when controlling for Somatic Focus, such that participants reported more pain when moderate stimulation was preceded by High- vs. Low-pain cues (t(22) = 7.64, p<0.001). This replicates and extends previous work [6], demonstrating that cue-based expectancies affect pain perception even when attentional focus varies on a trial-to-trial basis. Expectancy cues also affected Somatic Focus (Path *a*), such that participants performed better on the HDT when they expected High pain (t(22) = 2.36, p<0.05). However, controlling for Expectancy cue, Somatic Focus actually *reduced* pain reports (Path *b*), such that participants reported less pain when they performed correctly on the HDT (t(22) = −3.89, p<0.001). Finally, we found that expectancy effects on pain reports were indeed mediated by changes in somatic focus (*c – c’*; t(22) = −2.21, p<0.05), with a negative mediation effect, indicating that increases in somatic focus suppressed the pain-intensifying effects of high-pain expectancy [25]. Consistent with partial mediation and an overall suppression effect, we observed that the direct effects of Expectancy cues on pain (Path *c’)* were enhanced when controlling for Somatic Focus, as reported in Table 1. Together, these results indicate that expectancy-based enhancement of pain is not due to increases in attention to the body per se, and that focus on the HDT reduced pain.

**Table 1:** Mediation path coefficients^1^.

	a	b	c	c'	c-c'	d	e	e'	e-e'
**Across all trials**	0.09 (0.04)*	−0.14 (0.04)***	0.68 (0.09)***	0.7 (0.09)***	−0.005 (0.002)*	0.17 (0.04)***	−0.11 (0.04)**	−0.08 (0.04)*	−0.01 (0.003)**
**High pain expectancy**	n/a	−0.14 (0.04)***	n/a	n/a	n/a	0.25 (0.04)***	−0.20 (0.05)**	−0.18 (0.07)*	−0.01 (0.005)**
**Low pain expectancy**	n/a	−0.13 (0.06) *	n/a	n/a	n/a	0.11 (0.06) ⊥	−0.04 (0.05)	−0.002 (0.05)	−0.01 (0.005)
**Reward heat**	0.19 (0.06)***	−0.18 (0.06)***	0.62 (0.12)***	0.67 (0.12)***	−0.01 (0.004)*	n/a	n/a	n/a	n/a
**Reward visual**	0.03 (0.05)	−0.08 (0.06)	0.75 (0.09)***	0.74 (0.10)***	0.003 (0.002)	n/a	n/a	n/a	n/a

1 This table presents results from the multi-level mediation analyses presented in Figure 1. Values reflect mean path coefficients, with standard errors of the mean in parentheses.

*** = p<0.001;

** = p<0.01;

* = p<0.05;

⊥ = p = 0.058.

n/a values reflect the fact that those paths were not defined because we included only trials at one level of the independent variable for that path.

### Attention/Task Incentive Effects on Pain and Relationship to Somatic Focus: Mediation Model 2

**Figure 3:**
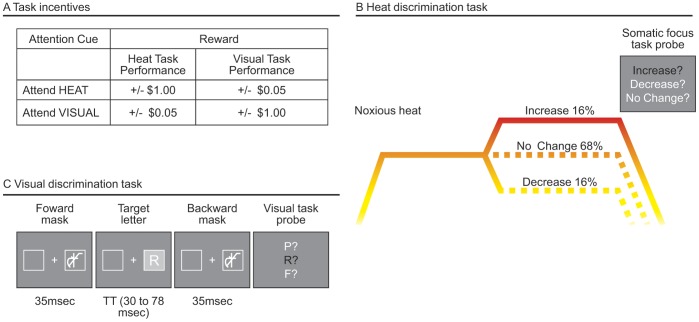
Somatic Focus manipulated with incentivized discrimination tasks. A. **Task incentives.** In order to provide incentive to follow the attention task cue (“Attend HEAT” or “Attend VISUAL”), we manipulated monetary payoffs, based on task performance. On Somatic Focus trials, participants were rewarded more for correct heat discrimination ($1.00) than visual discrimination ($0.05). Likewise, participants were penalized more for incorrect heat discrimination (-$1.00) than incorrect visual discrimination (- $0.05). On Visual Focus trials, the payoffs were reversed. Payoffs were determined from a subset of trials (20). The maximum additional reward compensation a participant could earn was $21 and the minimum was $0. B. **Heat discrimination task.** On 16% of trials the temperature increased by 1 degree, on 16% it decreased by 2 degrees (reductions are more difficult to detect based on piloting), and on the remaining trials there was no change. There were no changes in temperatures calibrated to elicit moderate pain (level 5), so we could test the effects of expectancy and attention on a constant, moderate level of pain stimulation. There was an equal distribution of increase, decrease, and no change trials on temperatures calibrated to elicit high and low levels of pain. In the no change trials, the stimulus intensity remained at the level determined to elicit a low (level 3) or high (level 7) amount of pain, based on the calibration procedure. For low and high pain, we used the levels 3 and 7, instead of 2 and 8, so that the decrease trials in the low-pain condition, and the increase trials in the high-pain condition would still be within the temperatures calibrated to elicit low and high pain (2 and 8, respectively). Participants reported no awareness of these contingencies in debriefing. C. **Visual discrimination task.** On each trial of the visual discrimination task, one of 20 consonants (excluding X) was briefly presented (for durations of 30 to 79 msec, depending on each participant’s calibrated performance) and masked immediately before and after by a mask constructed of overlapping letters. Letters and masks were presented directly to the left or right from a central crosshair. Participants had to determine which letter was presented on each trial.

Our second path model tested whether Task-Incentive cues affect pain reports, and whether this relationship was mediated by changes in somatic focus. We controlled for Expectancy Cue in all regressions. There was a significant effect of Task-Incentive cue on pain reports during moderate-pain trials (Path *e* in Figure 2A and Table 1; t(22) = −2.94, p<0.01), such that participants reported less pain when they were preferentially rewarded for performance on the HDT, relative to the VDT (see Figure 3A for task incentives). This effect corresponds to the main effect of Task-Incentive cue in Figure 2C. We found a significant effect of Task-Incentive cue on somatic focus (Path *d*; t(22) = 4.83, p<0.001), indicating that heat discrimination was better when subjects were preferentially rewarded for performance on that task. This effect corresponds to the main effect of Task-Incentive cue in Figure 2B, which additionally shows that participants performed at chance (dashed line) when the VDT was incentivized, but performed above chance when the HDT was incentivized. As before, somatic focus predicted reduced pain (Path *b*, which is statistically identical to Path *b* in *Mediation model 1*). Finally, the effect of Task-Incentive cue on pain was mediated by changes in somatic focus (*e – e’*; t(22) = −2.47, p<0.05). These results indicate that incentivizing the HDT reduced pain by increasing somatic focus.

### Interactions between Expectancy and Task Incentive Cue

**Figure 4:**
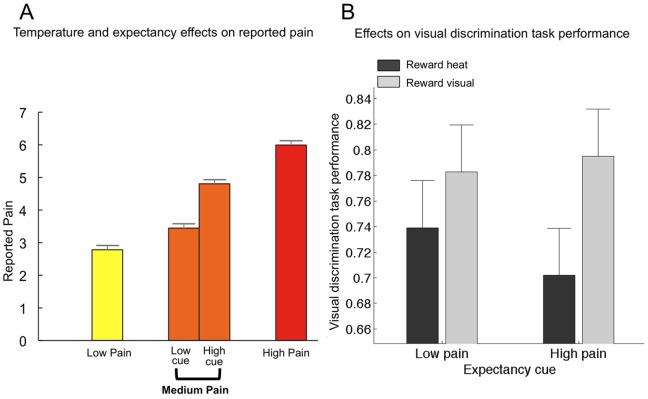
Main effects of Expectancy Cue on reported pain and Task-Incentive and Expectancy Cues on visual discrimination task performance. A. Expectancy effects on pain. There was a main effect of Expectancy Cue (high vs. low) on pain reports during the critical moderate pain stimulation trials, corresponding to Path *c* in our mediation model (p<.001) B. **Visual discrimination performance as a function of Task-Incentive Cue and Expectancy Cue.** There was a main effect of Visual Task Incentive Cue on visual discrimination task performance such that participants performed worse on the visual discrimination task when performance on the heat discrimination task was preferentially rewarded (p = .09 one-tailed). We found no cue-related interactions on visual discrimination task performance, though there was a trend towards reduced visual task performance with the combination of high pain expectancy and focus on the heat discrimination task (p = .11).

The mediation models presented above do not test for interactions between expectancy and task incentive, though we controlled for each cue type in each analysis. We therefore used a 2×2 repeated measures ANOVA to examine interactions between Expectancy Cue and Task-Incentive Cue on VDT performance, HDT performance, and reported pain on medium-pain trials. We found no cue-related interactions on VDT (t(22) = 0.65; p>0.5; see Figure 4 and Table 1), though there was a trend towards reduced VDT performance with the combination of high-pain expectancy and focus on the HDT (t(22) = −1.65; p = 0.11). There was a main effect of Visual Task Incentive Cue on Visual Task Performance such that participants performed worse on the visual discrimination task when performance on the heat discrimination task was preferentially rewarded (t(22) = −1.75 p = 0.09 one-tailed; see Figure 4B).

As shown in Figure 2B, there was a significant interaction between Expectancy Cue and Task-Incentive Cue on HDT performance in the expected direction, with a greater incentive-related increase in performance under high-pain expectancy (t(22) = −1.97, p = 0.03 one-tailed; see Table 1). Task-Incentive Cues favoring HDT performance increased HDT performance under High-pain expectancy (t(22) = 5.25, p<0.001), while effects under Low-pain expectancy were significant but weaker (t(22) = 1.98, p = 0.03 one-tailed). Finally, as shown in Figure 2C, there was a significant interaction between Expectancy Cue and Task-Incentive Cue on reported pain (t(22) = 2.16, p<0.05). Post-hoc t-tests indicated that Task-Incentive Cues modulated pain reports under High-pain expectancy (t(22) = −2.88, p<0.01), with reduced pain when incentives favored HDT performance, but did not affect reported pain under Low-pain expectancy (t(22) = −0.76, p>0.4). Main effects from these analyses were redundant with effects in the mediation model and are not reported; statistically significant results were obtained and agreed with the mediation model results in all cases. Overall, these results indicate that the pain-reducing effects of somatic focus were greatest under high-pain expectancy. Conversely, the pain-enhancing effects of pain expectancy were greatest when the VDT was incentivized.

### Cue-type Dependent Mediations

Because we observed significant interactions between Expectancy and Task-Incentive Cues, we assessed the mediation models described above separately for High-pain and Low-pain expectancy. The results and statistical details are presented in Table 1. In brief, we found that somatic focus negatively mediates Task-Incentive effects on pain only during High-pain cues. Thus, somatic focus was particularly helpful in reducing pain when pain expectancies were high. We also tested whether somatic focus mediated expectancy effects on pain separately for “Attend VISUAL” and “Attend HEAT” trials, and found that the enhancing effects of high-pain expectancy on somatic focus were observed only when attention was directed to the body.

## Discussion

In this study, we investigated the relationship between expectations about pain and attention to vs. away from the body, or somatic focus, on pain experience. Somatic focus was enhanced by two ‘top-down’ manipulations: Task instructions that incentivized discrimination of heat intensity, and cues that signaled increased pain intensity. It may be natural to think of pain expectancy as increasing attention toward the body, which thus enhances pain processing. However, our results suggest that this is not the case. Mediation analyses showed that pain expectancy exerts strong enhancing effects on pain experience that are not mediated by increased somatic focus. Rather, strikingly, somatic focus was associated with *reduced* rather than enhanced pain, partially suppressing the direct pain-enhancing effects of aversive expectancy. Thus, attention to the body promoted pain relief, and this was particularly true when pain expectancies were high.

These results both confirm prior work showing that pain perception assimilates towards expectations [2], [5], [6], [26], and show, for the first time, that expectations have effects that are strongly dissociable from the effects of attention to the body. This work also contributes new information to a long-standing debate on the potential effects of attention on pain. According to one set of studies, attention to the non-affective aspects of pain–as was required in our HDT–can reduce pain [27], [28]. However, other studies have reached the opposite conclusions, finding that focusing attention away from the body (e.g., via cognitive distraction) reduces pain [17], [29]–[31].

Several reviews on these effects have pointed out that the benefits of attention to vs. away from the body are strikingly inconsistent, with multiple studies finding either significant enhancement or significant reductions in pain, and others failing to find effects in either direction [12], [21], [32], [33]. According to these reviews, the discrepancies may be due to variable focus on the ‘hot,’ affective vs. the ‘cool’ sensory/discriminative aspects of pain (perhaps due to the lack of instruction sets in no-distraction conditions), variable levels of cognitive demand for internal vs. external tasks (and poor or absent measures of demand and performance), and possible interactions with time or pain intensity, with distraction being beneficial for brief or low-intensity pain, and body-focus–often accompanied by strategies such as ‘acceptance’ that minimize negative affective appraisal of pain–being beneficial for sustained and high-intensity pain.

Our experimental design included several unique features that allowed us to avoid the obvious confound of cognitive demand, and establish more precise relationships between expectancy, somatic focus, and pain. *First*, our tasks were chosen to load perceptual attention but not central executive resources, in order to allow both tasks to be performed simultaneously, and to minimize the pain-reducing effects of more cognitively-demanding tasks such as the N-Back [12]. Both the HDT and VDT involved change detection that occurred during only a few seconds of the 10-sec heat period, with the critical change lasting less than 1 sec, and both tasks were performed on each trial. Pain reports were consistent with those during calibration, which included no secondary tasks, suggesting that overall distraction effects as a result of cognitive demand were subtle if they occurred at all.

Second, we used performance measures to confirm that participants were attending where instructed on each trial. In contrast, many previous studies have compared conditions unmatched for cognitive demand–i.e., comparing counting backwards by threes with ‘pleasant imagery’ or even no instructions at all–and without performance measures that could assess the relative task difficulty or confirm control of attentional focus. As a result, the effects of attention on affective pain vs. sensory/discriminative aspects of body stimulation vs. distraction are confounded in the literature (see also [34]).

Third, the multi-level mediation approach we used allowed us to assess within-person relationships between experimentally manipulated variables, potential mediators, and pain report. This eliminates many sources of noise in between-person designs. In addition, we took an instrumental variable approach [35], independently manipulating both expectancy and somatic focus with task-incentive cues. With this approach, we focused on a mediation model that measured the effect of expectancy on somatic focus and pain. By manipulating somatic focus with task-incentive cues, we were able to confirm that somatic focus influences pain, rather than the reverse.

Thus, we believe the present study provides a relatively pure assessment of the effects of attention to vs. away from the body, independent of cognitive load effects. With this design, it appears plain that the effects of expectancy are not isomorphic with effects of attention to the body. However, painful experiences are multidimensional, and have both sensory/discriminative (“heat”) and nociceptive (“pain”) aspects. It is possible that expectancy cues increased attention to the *painfulness* of the heat or produced a kind of hyper-vigilance for pain, which increased pain experience, and only secondarily increased attention to the sensory/discriminative aspect of heat needed for HDT performance, which reduced pain. This explanation is consistent with other recent work suggesting that pre-stimulus brain activity thought to reflect attention to pain enhances pain experience [22]. It is also particularly plausible because non-affective sensory stimulation is known to antagonize nociception [36], [37], and the body-focus task we chose explicitly required cognitive judgments about the stimulus intensity and thus a focus on sensory/discriminative aspects of the heat. In this case, somatic focus as assessed by HDT performance indexes only one *kind* of attention (to heat intensity), and other kinds of attention (to pain or internal sensations of fear) might exacerbate pain.

Should the effects of expectancy be thought of in terms of a particular kind of attention (i.e., to pain) or another type of process (i.e., affective valuation)? This cannot be determined from the present behavioral results alone. However, an fMRI study using the same expectancy manipulation [6] found enhanced pre-stimulus activity with high pain cues in ventral striatum, and ventromedial prefrontal cortex, which have been associated with valuation processes but not with attention in the traditional sense (i.e., that elicited by cues that signal relevant visual events; [11]). Regardless of whether expectancy effects are labeled as a particular kind of attention or not, the empirical fact remains that they are dissociable from the effects of other kinds of attention to vs. away from the body. Future studies might productively investigate the distinction between focus on sensation vs. focus on pain in a secondary-task framework such as the one we employed.

These findings also suggest that the best cognitive strategies for mitigating pain might depend on the intensity of anticipated pain and the level of threat it entails. Though we did not find evidence for differential benefits of attention to the body as a function of time or stimulus intensity, as suggested by some earlier theorists [38], [39], our results suggest that the benefits of directing attention to the body *do* vary as function of pain expectancy. In the present study, the benefits of attention to the body were particularly important when pain expectancy was high. This may be because it is counterproductive to try to direct attention elsewhere (i.e., to the visual task) under high pain expectancy, or because somatic focus is effective at reducing threat-related processes elicited by high pain expectancy. In either case, intentionally focusing on the objective characteristics of the stimulus may mitigate distress associated with high pain expectancy by interfering with the affective components of pain processing.

Finally, our findings clarify a delicate interplay between threat, expectancy, and attention. According to the Affective Expectancy Model [40], emotional responses are generally assimilated toward expectancies, but may be biased away from expectations when individuals attend to precise characteristics of the stimulus [40], [41]. Our findings are generally consistent with this model, in that cue-based expectancy effects on pain were smaller when attention was directed toward the stimulus. However, our findings also suggest that an extension of the model is necessary, as expectancies can increase attention to the stimulus as well as directly influence affective experience. In our study, high pain cues caused increased somatic focus, which in turn predicted more acute somatic perception.

Together, these results indicate that expectancy-based enhancement of pain is not due to increases in attention to the body *per se*. It could be caused by attention to the specifically affective aspects of pain, or to affective valuation processes independent of the focus of attention. Whatever the case may be, focus on the site of pain (and particularly the heat intensity) may actually have beneficial effects on pain, particularly when high pain expectancies make focusing elsewhere more difficult. Finally, high pain cues had multiple, opposing effects on pain, both increasing pain directly and reducing pain through increased somatic focus. This finding suggests a greater variety of anticipatory processes than have previously been appreciated, whose underlying brain mechanisms and behavioral sequelae remain to be elucidated.

## Materials and Methods

### Ethics Statement

All participants provided written, informed consent in accordance with the Declaration of Helsinki. The ethics board of Columbia University specifically approved this study.

### Participants

Twenty-seven healthy, right-handed participants were enrolled. Four participants were excluded from the analyses: three due to equipment malfunction, and one who was unable to perform the HDT accurately, leaving 23 participants (16 female, mean age = 21.7 years, range = 18–45, sd = 5.51 years). Subjects reported no history of psychiatric, neurological, or pain disorders.

### Materials

Pain was elicited using a safe (i.e., non-damaging) thermal stimulus applied to the volar surface of the left forearm at temperatures selected to be painful but tolerable for each participant, up to 50°C (see “Thermal stimulation and pain ratings”, below). Stimuli were administered using a TSA-II Neurosensory Analyzer (Medoc, Inc.) with a 16×16 mm Peltier thermode (accuracy ±.1°C). Visual stimuli and task instructions were presented using PsychToolbox Version 3 [42] (http://psychtoolbox.org), a set of third-party scripts for Matlab (Mathworks, Inc., R2007b). Letter stimuli and masks subtended approximately 1 degree of visual angle. Auditory tones that served as expectancy cues were delivered at 500 Hz or 1000 Hz.

### Procedure

#### Thermal stimulation and pain ratings

All participants first underwent a calibration procedure, described in detail in [6]. This procedure allowed us to identify six sites on the forearm with similar nociceptive profiles, and to derive the individual participant’s dose-response curve for the relationship between applied thermal stimulation and reported pain. During calibration and the main experiment, participants rated pain experience after the termination of each noxious stimulus on a continuous 0–8 visual analog scale (0 = no sensation; 1 = non-painful warmth; 2 = low pain; 5 = moderate pain; 8 = maximum tolerable pain). Although this scale mixes painful and non-painful sensations, we judged its benefits to outweigh the costs, as it: a) provides implicit measures of pain threshold and tolerance; b) has good sensitivity within the painful range; and c) has good sensitivity to expectancy effects in a number of published experiments [5], [6], [43].

For each participant, the pain calibration allowed us to determine temperatures required to reliably elicit VAS ratings of low pain (rating = 3; *M* = 41.80°C, *SD* = 2.15), moderate pain (rating = 5; *M* = 43.72°C, *SD* = 1.76), and high pain (rating = 7: *M* = 45.7°C, *SD* = 1.718). These were used during the main task.

#### Pain expectancy cues

Participants were informed that auditory cues would predict high or low pain on every trial during the main experiment. We used two tones (500 Hz or 1000 Hz, 2 sec duration), counterbalanced across subjects. Prior to the experiment, participants performed a simple categorization task to ensure that they could differentiate between the tones. Participants classified them as predicting high or low pain, with >90% accuracy to continue with the experiment. No participants were excluded.

#### Task-Incentive cues

Participants were instructed that they would perform two tasks during thermal stimulation and that correct performance would always be rewarded, although the payoffs for correct performance on each task would vary across trials according to cues presented on the screen before each trial. They were told that the cue “Attend VISUAL” signaled that correct performance on the VDT would be rewarded with $1.00 in bonus money and incorrect performance would result in the loss of $1.00, whereas the gain/loss for correct/incorrect performance on the HDT would be only +/− $0.05. Conversely, the cue “Attend BODY” would signal the reverse contingencies, with $1.00 gain/loss based on the HDT, and +/− $0.05 based on the VDT (see Figure 3).

#### Visual discrimination task calibration

Before the main task, participants performed 50 trials of the VDT, with an adaptive staircase procedure designed to titrate the task timing so that each participant was approximately 75% accurate (chance performance was 25%). Each trial of the task began with a mask constructed of overlapping letters (35 msec; after [44]). Then, one of 20 consonants (excluding X, based on pilot testing) was presented for a variable interval, and masked immediately after by the overlapping-letter mask (35 msec). Letters and masks were presented directly to the left or right of a central fixation cross. Participants saw a subsequent *visual task probe* with three letters, one of which was the target letter, and indicated which letter had been presented with a button press. During this calibration block, the target presentation interval decreased if accuracy was greater than 75% in the last 4 trials and increased if it was less than 75%, with a step size of 20 ms divided by the square root of the trial number. Target presentation in the main task was based on each participant’s titrated interval timing, and ranged from 29.9 to 77.9 msec. (see Figure 3).

#### Main task

Over a series of 72 trials, participants experienced painful heat while performing two concurrent secondary tasks, the heat discrimination task (HDT) and the visual discrimination task (VDT). Each trial consisted of the following sequence, presented in detail in Figure 1B. First, as described above, the auditory pain expectancy cue signaled low- vs. high–temperature stimulation. Next, the task-incentive cue was presented, consisting of the words “Attend VISUAL” or “Attend HEAT” that signaled high-reward contingency on the visual- vs. heat-discrimination tasks, respectively. Following this cue, 10 sec of painful heat was delivered.

On an initial conditioning block (not included in the present analysis), low- and high-expectancy cues were always followed by low and high temperatures, respectively, to reinforce expectancies. On the remaining 60 trials, each cue was followed by either its predicted level (i.e. high or low intensity stimulation) or a single temperature calibrated to elicit medium pain. Specifically, medium heat was presented on 28 trials (48%) and high and low heat were presented on 16 trials each. Expectancy cues were fully crossed with task incentive cues on all trials, leading to equal numbers of trials under each condition. The medium-heat trials were of critical interest and are the main focus of our analyses.

During thermal stimulation, participants performed two concurrent tasks. In the HDT, participants detected a temperature change (increase, decrease, or no change) in the stimulus, which occurred at 4, 5, or 6 sec after heat onset (see Figure 3B). Importantly, there was no change on all of the medium-heat trials, so we could analyze only trials where temperature was constant. In the VDT, participants viewed a single masked letter, the presentation of which co-occurred with the temperature change. A constant target presentation interval was used for each participant (30 to 78 msec), based on the calibration procedure described above.

Visual and heat performance probes were presented after heat offset, prompting participants to indicate with a button-press which letter was presented and whether the heat increased, decreased, or did not change, respectively (see Figure 1B). Following the performance probes, participants rated the pain experienced on that trial.

#### Dependent measures and statistical analyses

As our first hypothesis concerned whether expectancy effects were mediated by somatic focus, we used multi-level mediation analyses implemented in custom Matlab software (freely available to the research community from http://wagerlab.colorado.edu). Linear relationships were assessed within-participants and subjected to group analysis for population inference in a mixed-effects model. In general, mediation tests assess whether the relationship between an initial variable (e.g., expectancy cue type) and outcome (pain report) is significantly reduced when controlling for a mediator (somatic focus, indexed by HDT performance; see below). The mediation test provides a stronger test of the relationships than simply testing the links individually (see, e.g., [18] for discussion). P-values were obtained for all tests in the mediation analyses using bias-corrected, accelerated bootstrapping [45] with 10,000 bootstrap samples. We used HDT performance as a measure of somatic focus rather than a composite of heat and visual task performance because the HDT provides the most direct measure of attention to the body, and because it was most sensitive to the expectancy and task-incentive cues.

The path model tested is diagrammed in Figure 1A. One mediation model was used to assess the effect of manipulated Expectancy cues (coded with 1, −1 values across trials) on pain report (Path *c*) and whether it was significantly mediated by somatic focus (coded with [1,−1] for correct or incorrect HDT performance on each trial). This entailed tests of Expectancy Cue effects on somatic focus (Path *a*), effects of Somatic Focus on pain report (Path *b*), and the mediation effect comparing Path *c* with the direct effect controlling for the mediator (Path *c*’). Task-Incentive Cue was controlled for when assessing each effect. A second model was used to assess the effects of Task-Incentive Cue (controlling for Expectancy Cue) on pain (Path *e*) and mediation by Somatic Focus (Paths *d* and *b*, with Path *b* identical to Path *b* above.) This analysis allowed us to assess the direct effects of each manipulation on pain and the indirect effect through somatic focus.

We supplemented the mediation analyses with simple repeated-measures ANOVAs to assess the main effects of Expectancy Cue, Task-Incentive Cue, and their interaction on pain reports and performance on both tasks. Significant interactions would imply that the effectiveness of Expectancy Cues on either outcome depend on Task Incentive, and/or vice versa, and thus the mediation results might only hold for *some* task conditions (e.g., only under body-focus incentives). Therefore, we tested each of the mediation models above *holding constant* (rather than controlling for) the effects of the other manipulated variable.
